# Optimization of a Web-Based Self-Assessment Tool for Preconception Health in People of Reproductive Age in Australia: User Feedback and User-Experience Testing Study

**DOI:** 10.2196/63334

**Published:** 2024-12-24

**Authors:** Edwina Dorney, Karin Hammarberg, Raymond Rodgers, Kirsten I Black

**Affiliations:** 1 Faculty of Medicine and Health, The University of Sydney, Susan Wakil Health Building, Western Avenue, Camperdown, 2050, Australia, 61 422-259-194; 2 Global and Women’s Health, School of Public Health, and Preventative Medicine, Monash University, Melbourne, Australia; 3 Robinson Research Institute School of Biomedicine, University of Adelaide, Adelaide, Australia

**Keywords:** technology, internet, eHealth, user experience, patient engagement, self-assessment tool, preconception, health communication

## Abstract

**Background:**

Good preconception health reduces the incidence of preventable morbidity and mortality for women, their babies, and future generations. In Australia, there is a need to increase health literacy and awareness about the importance of good preconception health. Digital health tools are a possible enabler to increase this awareness at a population level. The Healthy Conception Tool (HCT) is an existing web-based, preconception health self-assessment tool, that has been developed by academics and clinicians.

**Objective:**

This study aims to optimize the HCT and to seek user feedback to increase the engagement and impact of the tool.

**Methods:**

In-depth interviews were held with women and men aged 18‐41 years, who spoke and read English and were residing in Australia. Interview transcripts were analyzed, and findings were used to inform an enhanced HCT prototype. This prototype underwent user-experience testing and feedback from users to inform a final round of design changes to the tool.

**Results:**

A total of 20 women and 5 men were interviewed; all wanted a tool that was quick and easy to use with personalized results. Almost all participants were unfamiliar with the term “preconception care” and stated they would not have found this tool on the internet with its current title. User-experience testing with 6 women and 5 men identified 11 usability issues. These informed further changes to the tool’s title, the information on how to use the tool, and the presentation of results.

**Conclusions:**

Web-based self-assessment tools need to be easy to find and should communicate health messages effectively. End users’ feedback informed changes to improve the tool’s acceptability, engagement, and impact. We expect that the revised tool will have greater reach and prompt more people to prepare well for pregnancy.

## Introduction

### Overview

Preconception care aims to optimize the physical and psychological health of women and their partners prior to conception [1]. Good preconception health reduces the incidence of preventable morbidity and mortality for women and their babies [1,2]. In Australia, there is a need to increase awareness about the importance of good preconception health and access to preconception care [3]. This is evidenced by the high proportion of women entering pregnancy above a healthy weight, the low proportion of women taking folic acid before conception, and the low rate of good prepregnancy glycemic control in women with diabetes [4-7].

Barriers to the delivery of preconception care include inadequate knowledge in the community about the importance of optimal preconception health and a lack of presentation to health care providers for preconception assessment [8,9]. Additional barriers include women from low socioeconomic backgrounds having lower levels of functional health literacy [10]. This is associated with lower rates of consulting a health professional for preconception care and worse preconception health behavior [10,11]. Women also identify lack of time and living in rural and remote communities as prohibitive factors to seeking preconception care [12].

Evidence suggests that people of reproductive age are keen to learn about preconception health and adopt positive health behavior change before a pregnancy. Most women of reproductive age report a preference for internet-based information sources [13] and use technology to find information on pregnancy health [13,14]. Digital health tools, such as web-based self-assessment tools, are a promising medium to increase knowledge and awareness about preconception health among people of reproductive age across geographical locations [15,16]. This includes increased understanding of the benefits of good preconception health, the risks of poor preconception health, and the opportunities to improve health before conception [17,18]. Impacts of web-based preconception health self-assessment tools include reduced rates of preconception alcohol consumption, improved uptake of folic acid supplementation [19], and also act as a catalyst for clients to initiate discussions with health care providers [17].

### The Healthy Conception Tool

The Healthy Conception Tool (HCT) was an existing web-based preconception health self-assessment tool, developed by YourFertility, a Commonwealth-funded fertility health promotion program in Australia. The HCT asks questions about a person’s general and reproductive health from which people then get a personalized output of results. They are then encouraged to take the results to their doctor to discuss what they can do to optimize their health. The HCT contains a section for both women and men. The HCT was developed in 2017 in collaboration with academics, clinicians, and researchers at the Robinson Research Institute. Some feedback from users was used in the initial development of the HCT; however, formal usability testing was not performed. Exploring usability can identify issues with how people engage and interact with a tool, and the findings can be used to improve experience to maximize the effectiveness and efficiency of the tool [20].

As the HCT is a potential enabler to promote the importance of preconception health, particularly for people in rural and remote areas who face additional challenges in accessing health care, optimization and usability testing can enhance a tool for these populations. This paper describes the process taken to engage with communities using a person-centered approach to assess usability and optimize an existing web-based preconception health self-assessment tool for people in Australia.

## Methods

### Overview

**Figure 1.**
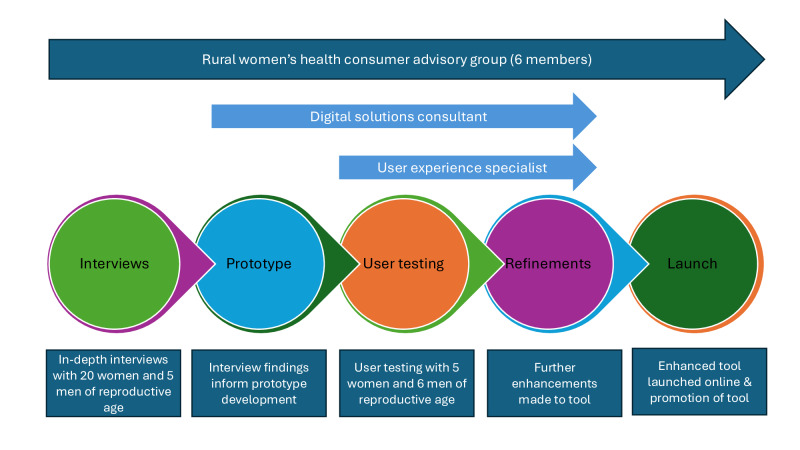
Process of optimizing a web-based self-assessment tool for preconception care.

In-depth user interviews and usability testing were used to optimize the existing HCT (Figure 1).

A rural women’s health consumer advisory group (RWH-CAG) was established to oversee this body of work and ensure person-centered design in all aspects. A total of 6 women aged 19‐30 years old, who resided in rural and remote locations in Australia, were selected from a pool of 17 applicants. Selected applicants had lived experience and demonstrated knowledge of issues around the access of sexual and reproductive services to rural Australian women, particularly in the areas of family planning and preconception care. The role of the RWH-CAG was to inform the study design and recruitment methods (Figure 1).

### Recruitment

People of reproductive age (18‐41 years old), residing in Australia, who could speak and read English, were eligible to participate in both the interviews and user-experience testing. People of all relationship statuses were eligible to take part.

Recruitment for interviews was via social media with advertisements targeting regional and remote community parent groups and noticeboards. Recruitment for user-testing was coordinated through a user-experience agency (Nomat Australia) with an Australia-wide registered pool of user-experience participants. The participants meeting the recruitment specifications listed above were sent an invitation to take part.

### Process

#### In-Depth Interviews

In-depth interviews to understand participants’ experiences of using the HCT were performed from February to September 2021. In particular, what features of the HCT were most likely to increase their knowledge and influence behavior change, and to explore ways in which the tool could be improved. Given COVID-19 quarantine restrictions, interviews were conducted via telephone or internet-based videoconferencing at the participants’ request. Before the interview, participants were required to complete the HCT. Interviews were recorded and transcribed, and data were analyzed by two researchers who have reproductive and public health expertise, using an inductive thematic approach [21,22]. Data were coded, manually without assistance of software programs, to identify consumer likes and dislikes across content and design features of the self-assessment tool. These findings were further discussed among all members of the research team, and agreement was reached on key domains and features for optimization

#### Prototype Development

A series of strategy, planning, and design workshops were held with a digital solutions consultant (Sentius Australia). These workshops aimed to develop creative and digital solutions for the new tool design, informed by the interview findings. A prototype was developed by Sentius and the research team, with a list of features identified for user testing.

#### Optimization of Prototype With User Testing

**Figure 2.**
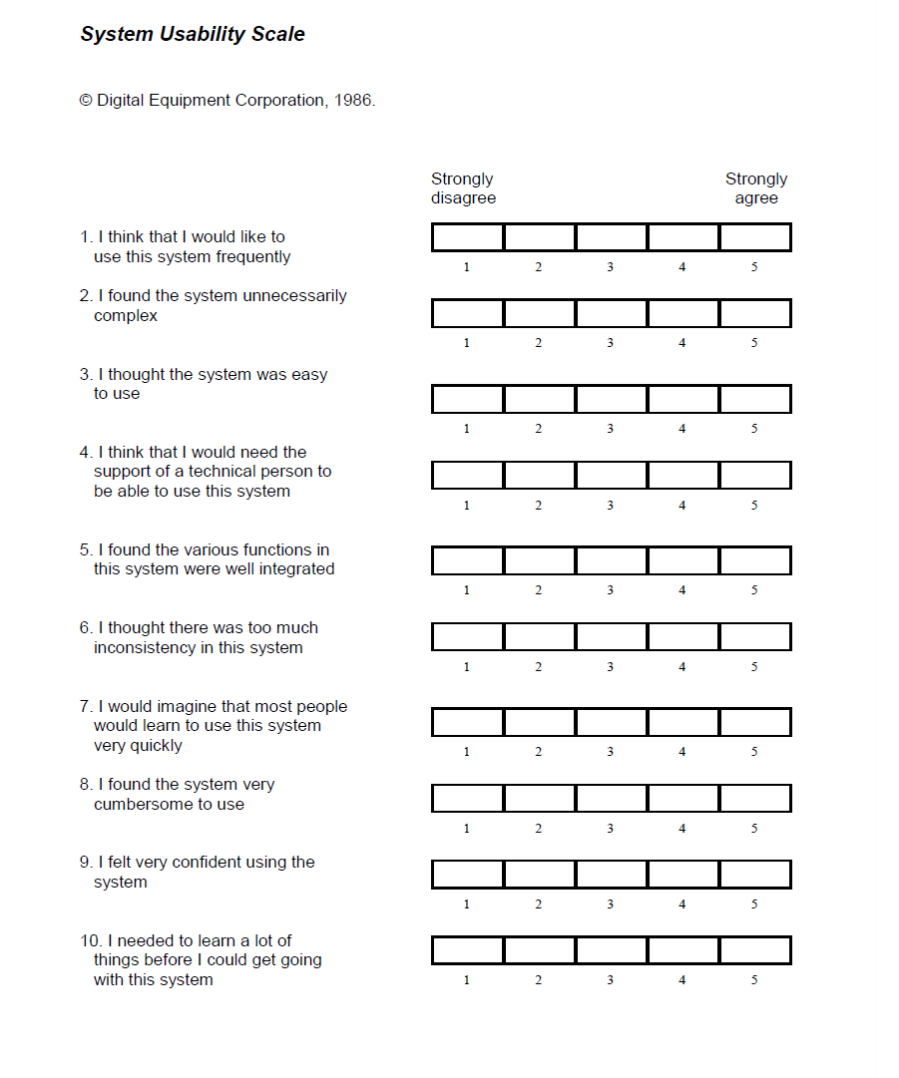
System Usability Scale [23].

The prototype underwent user-experience testing, conducted by a user-experience specialist to identify any usability issues and validate design changes that had been made from May to June 2022. Again, given the COVID-19 pandemic restrictions, the participants took part in a 1-hour videoconferencing call for user-experience testing. In this call, the participants completed the HCT in their chosen setting, to reflect their typical context. The participants shared their smart device screen so that their tool navigation could be directly observed. The participants completed the System Usability Scale (SUS) [23] and had the option to provide additional feedback on their experience. The SUS is a 10-item questionnaire to enable a standardized assessment of usability of an interface (Figure 2) and is an accepted tool to benchmark the usability of Digital Health Applications [24]. Usability problems were categorized as minor, moderate, or critical relating to the impact they had on tool engagement and completion (Table 1).

**Table 1.** Category definitions for identified usability issues.

Classification	Definition
Minor	Causes some hesitation or irritation
Moderate	Causes task failure for some users
Critical	Leads to task failure. Issue inhibits users from completing core tasks or may impact core business objectives

#### Prototype Refinement and Enhancements

User-experience testing results informed additional changes to the tool. Another round of planning workshops was held to decide on the final changes to the tool. These were tested amongst the research group for quality assurance.

#### Launch

When all changes were made, the enhanced tool was launched on the internet.

### Ethical Considerations

This study was approved the University of Sydney Human Research Ethics Committee (2020/430; June 22, 2020). Verbal consent was obtained prior to the interview and user testing. Recruitment for both processes ended when no new data were obtained, and previously identified themes were repeating. Participants were given a Aus $50 (US $37.50) VISA gift card in recognition of their time.

## Results

### Overview

In-depth interviews with 20 women and 5 men, and user-experience testing with 6 women and 5 men of reproductive age were performed. The participants included people planning and not planning a pregnancy in the next 12 months, from metropolitan, regional, and remote locations in Australia. The participant demographics are shown in Table 2.

**Table 2.** Participant demographics.

Demographics	Interview participants (n=25), n	User testing participants (n= 11), n
**Gender**		
Female	20	6
Male	5	5
**Pregnancy intention**		
Planner	10	6
Nonplanner	15	5
**Place of residence**		
Metropolitan	11	9
Regional and rural	14	2

### Interview Results

All participants completed the HCT prior to their interviews, using either a mobile device or laptop, with some completing the tool on both a mobile and laptop device. Attributes of the self-assessment tool, as identified from the interviews, are presented below.

#### General Features

Overall, participants were very positive about the HCT. Most participants liked that it was quick to use, taking on average 5 minutes to complete, and that it was a single source for multiple topics of pregnancy preparation.

#### Source Credibility

The participants expressed that it was important to know the tool was from a credible source. This was attributed to endorsement from reputable professional organizations such as academic institutions.

*I look for does it come from a trusted source, is it from the Royal Children’s Hospital website or is it from Breastfeeding Victoria’s website, not just a mummy blog or a website that has obvious ads or links to creams that they want to sell you.*

[P19, female, regional, nonplanner]

#### Target Audience

Participants liked that the tool was inclusive of partners with information for both men and women*.*

*I liked that it had a section for men and women, I think that’s really good, that men are included in it as well.*

[P6, female, regional, nonplanner]

#### Finding the Tool

The participants identified that a good tool is one that is intuitive to find on a simple web-based search. Almost all participants stated that they would not have found this tool on the internet with its current title.

#### Information Gained

The participants liked the links to further information, particularly the multiple and varied topics that were collated together.

*And I think the link that it had when you clicked on the little info icon, they took you to places, and there was like so much information in those places.*

[P6, female, regional, nonplanner]

#### Presentation of Results

The participants stated the presentation of their results was too general and that they wanted more personalized results. Consistently, the participants conveyed that the results must be meaningful to the individual to encourage behavior change.

#### User Experience

A total of 4 key domains for exploration in the user-experience testing were identified from the interviews:

1. User interface
2. Tool navigation
3. User experience
4. Results

A summary of the features for testing within these 4 domains is shown in Table 3. More details on the interview findings relating to these domains and features are available in Multimedia Appendix 1.

**Table 3.** Domains and features of a web-based self-assessment tool for preconception care to be assessed in usability testing.

Domain	Features to test
**User interface**	
Color	Color scheme
Images	Real life images Icons
**Tool navigation**	
Answer mechanisms	Free text Radio buttons Scroll bar Specified range
**User experience**	
Explanatory text	Explain how to answer the questions and what to do on completion
Sequence of questions	Sequence of questions for logical flow
Language	Title and text to avoid medical terminology Inclusive language and answer options Tone Appropriate health literacy
Accessibility of additional information	Display of additional information
**Results**	
Timing of results display within the tool	Give result with each individual question answered Give all results at the end of the tool
Visual display of results	Visual display of results with color coding system
Prioritized ordering and personalization of results	Prioritized display of results
Mechanism to receive and keep results	Email Print

### User-Experience Testing

All participants completed all questions in the prototype on their first attempt and responded well to its simplicity. The amount of information provided was considered appropriate, the supplied links were determined to be valuable, and information was perceived to be presented in a way that was easy to digest and use. The average SUS score was 91.82 (SD 4.62), which is a high score compared with an industry average in Australia of 68 (SD 12.5). The participants responded well to the given color scheme.

A total of 11 usability problems were identified, 4 moderate and 7 minor (Table 4).

Additional observations of significance were the impact of the organizational logos at the bottom of the tool. Without prompting, several participants commented on the organization logos and indicated that seeing this added credibility to the tool. New features to increase reach of the tool were also noted and included the option to share the tool with the user’s contacts.

**Figure 3.**
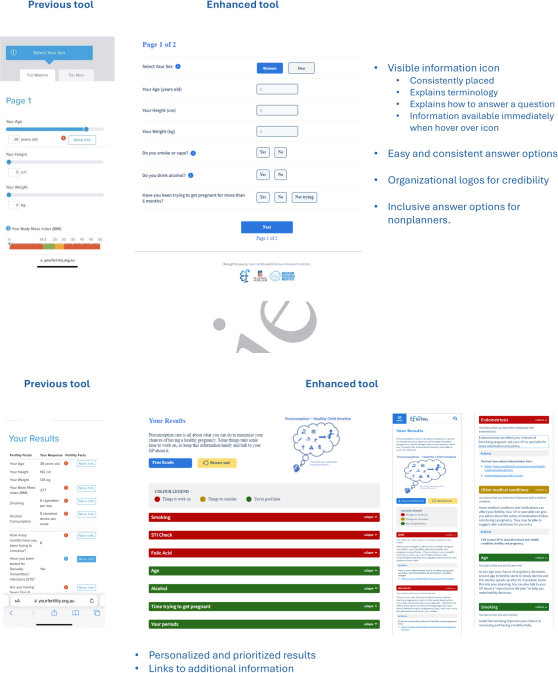
Enhancements to the self-assessment tool questions and results.

Additional refinements were made to the tool following user-experience testing to address the problems identified. Some key changes are shown in Figure 3.

**Table 4.** The findings on user-experience testing for a web-based self-assessment tool for preconception care.

Issue			Observation	Recommendation	Severity
**Results**					
Presentation of results—traffic light system colours			Consistently, the color coding on the results page was not obvious to participants. Particularly, the use of orange instead of red was misunderstood. “*I don’t understand why they’ve done that...maybe change the choice of colours to red, yellow and green? I would be fine with red*” (P36, male, planner, metropolitan)	Update to green, orange, red and introduce descriptive headings within the results page.	Moderate
Grouping preference			Consistently, participants indicated that they preferred the layout of the second version of the results page (order as per traffic lights). Specifically, participants suggested that grouping results led to increased understanding of the content grouping. *“Now it makes more sense to see the colours categorised in groups.”* (P28, female, non-planner, metropolitan)	Implement grouping of results as the standard.	Moderate
**User experience**					
Help icon: visibility			The help icon was deemed to be of value to participants, however it was not immediately obvious to all participants. The visual hierarchy needs to be increased, in order to prompt a higher use rate. *“No, I don’t recall seeing these help icons, that would have been good to know for this question [fertile window].”* (P26, female, non-planner, metropolitan)	Consider making the help icon 15% larger and changing the color to HEX #14c797 to increase prominence.	Moderate
Answer clarification: smoking and alcohol			For both the smoking and alcohol use questions, some participants answered ”no” despite describing infrequent use. This indicated that just having 2 possible answers causes misreporting in some instances. “*It’s a tad confusing, the smoking and drinking. It’s a yes and a no answer but there’s a lot of gradations in there...someone might have two glasses a week.”* (P34, male, planner, metropolitan)	Include help icon to explain how to answer the question.	Moderate
** Question clarification**					
Chemicals			Participants often displayed confusion as to what kind of chemicals this question referred to. “*I don’t know much about chemical exposure...maybe a Tefal pan? I’m not sure. That would have been a question I could use some extra information for.”* (P28, female, nonplanner, metropolitan)	Include help icon text to expand on the types of household chemicals that could impact fertility.	Minor
STI check			On occasion, participants expressed surprise at the inclusion of the STI check question. Specifically, participants did not feel it was relevant to them if they were in a long-term relationship. *“Why is this question relevant to pregnancy?”* (P30, male, planner, metropolitan)	Include help icon text to explain the relationship between STI checks and future fertility. Reiterate STIs can be asymptomatic	Minor
Prescription meds			Participants indicated confusion around the types of prescription medication that would be included here. *“I’m on the mini [contraceptive] pill, but I wouldn’t tick yes here.”* (P27, female, planner, metropolitan)	Add help icon text to explain prescription medications	Minor
Folic acid			Folic acid prompted some additional discussion from participants, with questions raised about what it was, the dose required, and the function it served. “*I would answer no to taking folic acid...I haven’t even heard that mentioned. Now I wonder if that is important or not...I am not sure what it is.”* (P33, female, planner, regional)	Add help icon text to give a brief explanation of the importance of folic acid. Mention that folic acid is included in pre-natal supplements.	Minor
Results page: BMI information			Participants indicated that the BMI information required additional context to be more effective. “*The BMI information is good, but you just want a quantified benefit. You always hear about eating healthy and being healthy...it just becomes noise. Unless there’s a real benefit, what’s the point?”* (P30, male, planner, metropolitan)	Expand results section to include specific statistics connecting BMI with fertility. Include more targeted exercise suggestions.	Minor
Language: male version			Throughout the text on the male pages, some of the wording occasionally confused male participants. Some male participants did not feel that this was relevant to them and questioned whether the results were actually targeted towards the male user.	Tailor copy for participant gender.	Minor
**User interface**					
Image: sperm regeneration			Without being prompted, male participants consistently drew attention to the sperm regeneration fact at the top of the results page. Participants indicated that this statistic was new to them, and that they valued having this information included.	On the results page, incorporate this information within the main content.	Minor

## Discussion

### Principal Findings

This work explored what features of a web-based self-assessment tool for preconception care are important to people of reproductive age. In particular, what features will increase engagement and completion of the tool, and what are the best ways to present the results and information so that a user will act upon them.

Our findings showed that participants value a tool that is intuitive to find in a web-based search and is quick and simple to use. This is consistent with findings of other eHealth modalities, where ease of use and simplicity is a determinant in user engagement [25,26]. Clear information about what each question is asking was important to maintain engagement in the tool. For questions that were not intuitive, or required explanation, an information icon was placed adjacent to the question text, in a different color to emphasize its presence. The information was visible by hovering over the icon, as participants indicated they did to want to be directed to an additional page, as this interfered with user experience. Having information presented in a way that is easily understood by the user has been identified as a key quality indicator of web-based health sites [27].

A key finding in both the interviews and user-experience testing was the need to have information clearly presented and easy to digest at an appropriate health literacy level. The International Reproductive Health Education Collaboration recently devised recommendations for developing and implementing tools to improve fertility literacy [28]. This included the recommendation to understand the target population and include user perspectives when developing education tools. In both the interviews and user-experience testing. there was a balance of planners and nonplanners (those not planning a pregnancy in the next 12 mo) to ensure the information presented was accessible and relevant to all people of reproductive age regardless of pregnancy intention. This also led to some key changes including to the title of the tool. Almost all of the interview participants would not have found the tool on the internet, and as such the tool was renamed with a plain English title as informed by participants of “Healthy you, Healthy baby*.”*

Another key feature for users was the personalized and ordered presentation of tool results. Results were grouped into categories that required the user’s attention and action. These were color-coded to convey visual priority and accompanied by explanatory text, in a positive tone to complement the color scheme.

The participants expressed the importance of knowing that a source is credible, and this is acknowledged by the inclusion of logos from trusted organisations. Trust in eHealth sites is a recognized determinant of user engagement with web-based health information sources [29]. Accreditation or endorsement with recognized logos from reputable institutions can increase trust in a platform [30].

eHealth platforms have been shown to be effective in improving user health knowledge, behavior change, and health outcomes [31,32]. Several eHealth platforms have been shown to be effective for the communication of preconception health information [33-35]. A web-based app for people with sickle cell disease and sickle cell trait providing information about prepregnancy health was found to be acceptable and usable and increased consumer knowledge [33]. An eHealth lifestyle coaching program for women prior to pregnancy has been shown to increase healthy eating behaviours [34,35].

Studies have suggested improvement in eHealth intervention designs to increase their effectiveness [32]. This includes adopting a holistic approach to promote user engagement [36]. Our approach, informed by a rural women’s health consumer advisory group, of in-depth interviews followed by user-experience testing enabled a detailed understanding of our target audience needs and expectations. The opportunity to test the consumer, informed prototype, and validated design has delivered an enhanced tool for people of reproductive age in Australia.

### Strengths

The use of both interviews and user-experience testing techniques are a strength of this study and provided additional iterations to enhance the self-assessment tool. The involvement of the RWH-CAG from conception to completion of this project also ensure a person-centred approach.

### Limitations

The tool was only explored by people who can speak and read English, and therefore does not capture the preferences of people from culturally and linguistically diverse backgrounds. As these populations can face challenges with access to care, this is a priority area for future research. This study used the SUS as an instrument within the usability testing. Further enhancements may be achieved by using additional tools such as Neilsen’s guidelines in combination with the SUS [37].

### Conclusion

As a web-based tool, “Healthy You, Healthy Baby” has the potential to improve knowledge among people of reproductive age about the importance of optimal preconception health, including those who experience health inequities, such as women and men in rural and remote areas. The tool can be adapted to other priority populations, including people from culturally and linguistically diverse backgrounds to further improve the delivery of preconception care.

## Supplementary material

10.2196/63334 Multimedia Appendix 1 Interview findings outlining the domains and features for testing of the online self-assessment tool for preconception care.

## Abbreviations

HCT: Healthy Conception Tool
RWH-CAG: rural women’s health consumer advisory group
SUS: System Usability Scale
