# Supplementary material for: Optimization of a Web-Based Self-Assessment Tool for Preconception Health in People of Reproductive Age in Australia: User Feedback and User-Experience Testing Study
Source: JMIR Hum Factors. 2024 Dec 24;11:e63334. doi: 10.2196/63334 (PMC11693785; doi:10.2196/63334)
Supplement: Multimedia Appendix 1 [file humanfactors-v11-e63334-s001.docx]

**Table S1. Domains and features of an online self-assessment tool for preconception care to be assessed in usability-testing**

| **Domain** | **Interview Findings** | **Features to test** |
| --- | --- | --- |
| **User interface** | | |
| **Colour** | *“I think the page itself was – it needed a bit of colour, maybe a better design…. I think if I had just opened it up and found it online, just purely looking at the design of it, I wouldn’t have taken it as seriously.”* **(Female, planner, metropolitan)** | Colour scheme |
| **Images** | *“I think picture, I always like pictures, I’m a visual person so I think pictures would have been good as well.”* **(Female, planner, regional)** | Real life images |
|  |  | Icons |
| **Tool Navigation** | | |
| **Answer mechanisms** | *"Like the first question was your age, and so I automatically went to the box to type in my age, I didn't pull the – this thing, what do you call it?"* **(Female, non-planner regional)** | Free Text  Radio Buttons |
|  | *I wish you would get rid of the slide-y bar thing. That big slide-y bar.* (**Female, non-planner, regional)** | Scroll bar |
|  | *"So, I think that weight range was a little bit intimidating"* **(Female, planner metropolitan)** | Specified range |
| **User experience** | | |
| **Explanatory text** | *“I think one of the things I did sort of find was that there wasn't a lot of operational or explanatory information on how to do the survey."* **(Female, non-planner, regional)** | Explain how to answer the questions |
|  | *“And I think almost a message about, "It's really important to go and seek the advice of your GP in conjunction with this."* **(Female, non-planner, metropolitan)** | Guidance at tool completion |
|  | *"And you could also maybe – maybe putting something in about that you can go back into the tool. Because, sometimes when you do surveys, you can't access them again."* **(Female, non-planner, regional)** | Guidance text to return to the tool |
| **Sequence of questions** | *"The topics were quite relevant; I just did feel they did jump a little bit. Like the individual topics were quite relevant, but there was no flow. It didn’t flow as nicely as it possibly could."* **(Female, planner, rural)** | Sequence of questions for logical flow |
| **Language** | *"Getting pregnant, wanting to have a baby. It'd be even more sort of colloquial, kind of terms…. Yeah, I would probably not even put conception in that."* **(Female, non-planner regional)** | Title and text to avoid medical terminology |
|  | *"It could be a little bit more relaxed and a little bit more less formal, because it’s not a doctor giving you your test results”* **(Female, planner, rural)** |  |
|  | *"Other than that question not really including anyone who’s trying to get pregnant through IVF or in that sort of, I guess, non at home mode."* **(Female, planner, rural)** | Inclusive language and answer options |
|  | *“However, I did find that some of the language, especially around BMI et cetera was a little bit judge-y.”* **(Female, planner, rural)** | Tone |
|  | *"I think it’s really important to have it in normally words. I know it feels more professional and more important if it’s in proper academic words, but most people don’t understand that and they don’t understand the concept. And if you’re too busy trying to read the big words, you don’t actually get the full story."* **(Female, planner, rural)** | Appropriate health literacy |
| **Accessibility of additional information** | *“…there was a little alert that came up, but it didn’t really come up as a – I didn’t really think it was enough of a warning, it was just something that you could very easily skim over.”* **(Female, planner, rural)** | Display of additional information |
|  | I think something explaining if you get a green tick, you can click on it for more, to clarify the information. Or, if you get a red explanation, click on it and it will help you find out more info about how you can change things or whatever. **(Female, non-planner regional)** | Placement of information icon |
| **Results** | | |
| **Timing of results display within the tool** | *"That was the overwhelming sense I was getting, going through it. It was like, "I keep seeing red; that means I'm not going to be able to do it."* **(Female, non-planner, metropolitan)** | Give result with each individual question answered |
|  | *"I like seeing them just on the screen, straight away. You know when you’ve finished something, I like to see the results straight away."* **(Male, planner, metropolitan)** | Give all results at the end of the tool |
| **Visual and text display of results** | *“...then seeing a green tick versus a red exclamation mark, I find that really quite confronting, because that could lead you down, just from a visual perspective, really lead you down the path of green means good, read means bad.”* **(Female, non-planner, metropolitan)** | Visual l display of results in traffic light system (orange not red colour) |
|  | *"But yeah, I think definitely highlighting it, whether it be through a traffic light system, or another way, yeah."* **(Female, non-planner regional)** | Explanation of results no colour coding system. |
| **Prioritised ordering and personalisation of results** | *"Yeah, I think because it didn’t - like if I remember correctly, it doesn’t say any areas of concern. So even though, for example I didn't drink alcohol, that result is given kind of equal weighting to me saying that I don't exercise. So I can't kind of gage which one's good or bad."* **(Female, non-planner, regional)** | Prioritised display of results |
|  | *"Like a little bit of more prioritising of what is and isn’t important."* **(Male, non-planner, metropolitan)** |  |
|  | *"I'm not sure if it gave me any specific details about me personally. I just thought it was very generalised."* **(Female, planner, regional)** |  |
| **Mechanism to receive and keep results** | *"Being able to download the information was handy for later reference, or even to take to your pre-conception appointment kind of thing – that was good, yes. I like the way it was listed in table format."* **(Male, non-planner, metropolitan)** | Email  Print |

| **Domain** | **Interview Findings** | **Features to test** |
| --- | --- | --- |
| **User interface** | | |
| **Colour** | *“I think the page itself was – it needed a bit of colour, maybe a better design…. I think if I had just opened it up and found it online, just purely looking at the design of it, I wouldn’t have taken it as seriously.”* **(P16, female, planner, metropolitan)** | Colour scheme |
| **Images** | *“I think picture, I always like pictures, I’m a visual person so I think pictures would have been good as well.”* **(P9, female, planner, regional)** | Real life images |
|  |  | Icons |
| **Tool Navigation** | | |
| **Answer mechanisms** | *"Like the first question was your age, and so I automatically went to the box to type in my age, I didn't pull the – this thing, what do you call it?"* **(P6, female, non-planner regional)** | Free Text  Radio Buttons |
|  | *I wish you would get rid of the slide-y bar thing. That big slide-y bar.* **(P19, female, non-planner, regional)** | Scroll bar |
|  | *"So, I think that weight range was a little bit intimidating"* **(P15, female, planner metropolitan)** | Specified range |
| **User experience** | | |
| **Explanatory text** | *“I think one of the things I did sort of find was that there wasn't a lot of operational or explanatory information on how to do the survey."* **(P6, female, non-planner, regional)** | Explain how to answer the questions and what to do on completion |
| **Sequence of questions** | *"The topics were quite relevant; I just did feel they did jump a little bit. Like the individual topics were quite relevant, but there was no flow. It didn’t flow as nicely as it possibly could."* **(P11, female, planner, rural)** | Sequence of questions for logical flow |
| **Language** | *"Getting pregnant, wanting to have a baby. It'd be even more sort of colloquial, kind of terms…. Yeah, I would probably not even put conception in that."* **(P6, female, non-planner regional)** | Title and text to avoid medical terminology |
|  | *"Other than that question not really including anyone who’s trying to get pregnant through IVF or in that sort of, I guess, non at home mode."* **(P4, female, planner, rural)** | Inclusive language and answer options |
|  | *“However, I did find that some of the language, especially around BMI et cetera was a little bit judge-y.”* **(P11, female, planner, rural)** | Tone |
|  | *"I think it’s really important to have it in normally words. I know it feels more professional and more important if it’s in proper academic words, but most people don’t understand that and they don’t understand the concept. And if you’re too busy trying to read the big words, you don’t actually get the full story."* **(P11, female, planner, rural)** | Appropriate health literacy |
| **Accessibility of additional information** | *“…there was a little alert that came up, but it didn’t really come up... it was just something that you could very easily skim over.”* **(P10, female, planner, rural)** | Display of additional information |
| **Results** | | |
| **Timing of results display within the tool** | *"That was the overwhelming sense I was getting, going through it. It was like, "I keep seeing red; that means I'm not going to be able to do it."* **(P17, female, non-planner, metropolitan)** | Give result with each individual question answered |
|  | *"I like seeing them just on the screen, straight away. You know when you’ve finished something, I like to see the results straight away."* **(P23, male, planner, metropolitan)** | Give all results at the end of the tool |
| **Visual display of results** | *"But yeah, I think definitely highlighting it, whether it be through a traffic light system, or another way, yeah."* **(P6, female, non-planner regional)** | Visual display of results with colour coding system. |
| **Prioritised ordering and personalisation of results** | *"Yeah, I think because it didn’t - like if I remember correctly, it doesn’t say any areas of concern. So even though, for example I didn't drink alcohol, that result is given kind of equal weighting to me saying that I don't exercise. So I can't kind of guage which one's good or bad."* **(P1, female, non-planner, regional)** | Prioritised display of results |
|  | *"Like a little bit of more prioritising of what is and isn’t important."* **(P24, male, non-planner, metropolitan)** |  |
|  | *"I'm not sure if it gave me any specific details about me personally. I just thought it was very generalised."* **(P5, female, planner, regional)** |  |
| **Mechanism to receive and keep results** | *"Being able to download the information was handy for later reference, or even to take to your pre-conception appointment kind of thing – that was good, yes. I like the way it was listed in table format."* **(P21, male, non-planner, metropolitan)** | Email  Print |
